# *Notes from the Field*: Characteristics of Meat Processing Facility Workers with Confirmed SARS-CoV-2 Infection — Nebraska, April–May 2020

**DOI:** 10.15585/mmwr.mm6931a3

**Published:** 2020-08-07

**Authors:** Matthew Donahue, Nandini Sreenivasan, Derry Stover, Anu Rajasingham, Joanna Watson, Andreea Bealle, Natasha Ritchison, Thomas Safranek, Michelle A. Waltenburg, Bryan Buss, Jennita Reefhuis

**Affiliations:** ^1^Epidemic Intelligence Service, CDC; ^2^Nebraska Department of Health and Human Services; ^3^CDC COVID-19 Emergency Response Team; ^4^Division of Global Health Protection, Center for Global Health, CDC; ^5^Western States Division, National Institute for Occupational Safety and Health, CDC; ^6^Dakota County Health Department, Dakota City, Nebraska; ^7^Division of State and Local Readiness, Center for Preparedness and Response, CDC.

Coronavirus disease 2019 (COVID-19) has been reported nationwide among meat processing facility workers (*1*). In late April 2020, through flyers and text messages, workers at a Nebraska meat processing facility were invited by the facility, in partnership with the Nebraska Department of Health and Human Services, to be tested for current SARS-CoV-2, the virus that causes COVID-19, at their worksite, free of charge. Specimens were analyzed using reverse transcription–polymerase chain reaction (RT-PCR) by a contracting laboratory. This investigation was determined by CDC to be public health surveillance.* Among 1,216 Nebraska-resident meat processing facility workers tested, 375 (31%) had positive results. During May 8–25, case investigators attempted to interview the 349 workers who had positive test results and available phone numbers; five refused, 99 were not reached after five attempts, and four did not report symptom status, leaving 241 (69%) of the attempted interviews for analysis.

Among the 241 interviewed workers, 57% were male, the median age was 41 years (range = 18–76 years), and 46% were Hispanic (Table). Approximately one third (78; 32%) of respondents reported no symptoms. Among the 163 symptomatic respondents, two were hospitalized, and no deaths were identified. Workers were queried about exposures during the 14 days before symptom onset (*2*) or before testing if they were asymptomatic. Close contact^†^ with a visibly ill person (or person with diagnosed COVID-19) at work was reported by 70 (29%) workers; the most frequently reported close contact locations were production areas (74%) and cafeteria/break areas (51%). Among 167 persons who worked in the 14 days preceding symptom onset or testing, approximately half (46%) worked on the conveyor belt in harvesting (i.e., stunning, slaughtering, eviscerating, and halving), processing (i.e., cutting, preparing, and packaging), and rendering (i.e., converting waste animal materials into usable products), where they were in close proximity (<4 ft [<1.5 m]) to others. Most (88%) workers reported using a private vehicle rather than carpooling (11%) to get to work. Although most (87%) reported always having their temperature checked upon entry to work, fewer (41%) reported always being asked about symptoms. Nearly three quarters of workers (73%) reported having a flexible medical leave policy allowing for time off if needed. Approximately one half of workers reported living in a single-family home (53%), with a median household size of three persons (range = 1–13). Thirty of 235 (13%) workers reported close contact with a visibly ill person (or a person with diagnosed COVID-19) outside of work. Limitations of this analysis include the absence of a comparison group and that only persons who participated in testing, had positive test results, had contact information, answered the telephone, and agreed to be interviewed were included.

**TABLE:** Demographic, clinical, household, community and occupational characteristics of 241 meat processing facility workers with confirmed SARS-CoV-2 infection — Nebraska, April–May 2020

Characteristic (no. with available information)	No. (%)*
**Sex (237)**	
Male	136 (57)
Female	101 (43)
**Age group, yrs (238)**	
Median age, yrs (range)	41 (18–76)
18–29	41 (17)
30–39	70 (29)
40–49	44 (18)
50–59	55 (23)
≥60	28 (12)
**Ethnicity (210)**	
Hispanic	97 (46)
Non-Hispanic	113 (54)
**Reported preferred language^†^ (220)**	
Spanish	75 (34)
English	56 (25)
Somali	54 (25)
Other	35 (16)
**Underlying health conditions (238)**	
None	195 (82)
Any^§^	43 (18)
Diabetes^¶^	21 (9)
Cardiovascular disease^¶^	15 (6)
Lung disease	8 (3)
**Signs and symptoms associated with illness (241)**	
None	78 (32)
Any	163 (68)
Headache	106 (44)
Fatigue	85 (35)
Measured or subjective fever	82 (34)
Myalgia	82 (34)
Lost taste or smell	77 (32)
Cough	59 (24)
Sore throat	57 (24)
Chills	52 (22)
Median illness duration, days (range)	11 (<1–31)
**Outcome (241)**	
Hospitalized	2 (1)
Died	0 (0)
**Smoking habits (236)**	
Never smoker	177 (75)
Former smoker	46 (19)
Current smoker	13 (6)
**Occupational exposures**	
Close contact** with ill person at work (241), no. (% of total)	70 (29)
Production areas, no. (% of 70)	52 (74)
Cafeteria/Break areas, no. (% of 70)	36 (51)
Locker room, no. (% of 70)	30 (43)
Entry/Exit, no. (% of 70)	28 (40)
Other, no. (% of 70)	12 (17)
**Worked 2 wks before symptoms or test^††^ (237)**	**167 (68)**
**Occupational role**^§§^ (167)	
Harvesting (stunning, slaughtering, eviscerating, halving)^¶¶^	27 (16)
Chilling	12 (7)
Processing (cutting, preparing and packaging meat products)^¶¶^	91 (54)
Rendering (converting waste animal materials into usable products)^¶¶^	3 (2)
Material handling	21 (13)
Administrative support/Other	16 (10)
Commute to work*** (167)	
Carpool	19 (11)
Private car	147 (88)
Other	5 (3)
**Wore a face covering or mask at work (157)**	
Always	142 (90)
Sometimes	8 (5)
Never	7 (4)
**Aware of flexible leave policy (164)**	
Yes	120 (73)
No	18 (11)
Don’t know	26 (16)
**Temperature checked at work entry (160)**	
Always	139 (87)
Sometimes	9 (6)
Never	12 (8)
**Symptoms checked at work entry (162)**	
Always	66 (41)
Sometimes	17 (10)
Never	79 (49)
**Household and community characteristics**	
**Household size, no. of persons including interviewed worker (228)**	
Median (range)	3 (1–13)
1	38 (17)
2	63 (28)
3	46 (20)
4	36 (16)
5	22 (10)
≥6	23 (10)
**Home type (233)**	
Single-family home	124 (53)
Apartment	99 (42)
Mobile home or other	10 (4)
**Household member works outside home (234)**	
No one else worked outside home	119 (51)
Household member works outside home^†††^	115 (49)
Same facility, no. (% of 115)	83 (72)
Other food or manufacturing facility, no. (% of 115)	11 (10)
Health care, long-term care facility, school, or child care, no. (% of 115)	9 (8)
Other, no. (% of 115)	18 (16)
**Household member ill or has positive test result for SARS-CoV-2 (236)**	
Household member ill or has positive test result for SARS-CoV-2 before or after worker	63 (27)
**Community exposures**	
Close contact^††^ with ill person outside work, including ill household members (235)	30 (13)
Not sure about close contact with ill person outside work (235)	22 (9)
Used public or shared transportation (236)	12 (5)
Household member at school or child care facility (238)	3 (1)
Attended social gathering of >10 persons (234)	3 (1)

* Because of missing data, categories might not sum to total.

^†^ Information on preferred language was included instead of race because more complete and detailed information was available for this diverse population. Other languages include Burmese, Cambodian, French, Karen, Lao, Malay, Oromo, Romanian, Tigrinya, and Vietnamese.

^§^ Other underlying conditions that were asked about and reported infrequently include: renal conditions, liver conditions, autoimmune disorders, neurologic disorders and other chronic conditions.

^¶^ Six workers reported underlying cardiovascular disease and diabetes.

** Close contact was defined as being within 6 ft (2 m) of an ill person for ≥10 minutes in the 2 weeks preceding symptom onset or testing.

^††^ No information is available on why the workers who did not go to work in the 14-day period were absent.

^§§^ Six workers had multiple occupational roles.

^¶¶^ Those working on the belt in harvesting, processing, and rendering were considered to work in proximity (<4 ft [<1.5 m]) to one another.

*** Four workers used multiple modes of transportation to get to work.

^†††^ Six workers had two household members who worked outside the home in different industries. It is possible that multiple household members who worked in the same plant are included in this study.

Reducing workplace exposures is crucial for preventing COVID-19 among meat processing facility workers. Despite broad availability of a flexible medical leave policy and fever screening, approximately one third of workers included in this investigation reported close contact with an ill person at work, which supports the need for symptom screening^§^ in addition to fever screening and ongoing access to testing. Fewer workers reported contact with an ill person outside work; risk factors such as crowded living conditions and shared transportation were reported infrequently. Approximately one third of workers with COVID-19 were asymptomatic, underscoring the limitations of relying on symptom or fever screening alone, particularly because asymptomatic persons with COVID-19 potentially contribute to transmission (*3*,*4*). That nearly one half of interviewed workers worked in close proximity to others highlights the need for physical barriers between workers, physical distancing throughout the facility (especially locations prone to crowding, such as production areas and cafeterias or break areas), and consistent and correct use of masks to reduce transmission in the workplace^¶^ in this critical industry (*5*,*6*).

## Footnotes

* U.S. Department of Health and Human Services, Title 45 Code of Federal Regulations 46, Protection of Human Subjects. https://www.ecfr.gov/cgi-bin/retrieveECFR?gp=&SID=83cd09e1c0f5c6937cd9d7513160fc3f&pitd=20180719&n=pt45.1.46&r=PART&ty=HTML.

† Close contact was defined as being within 6 feet (2 m) for ≥10 minutes in the 2 weeks preceding symptom onset or testing.

§ Symptom screening should include some of the wide range of symptoms that persons with COVID-19 have reported (e.g., fever, cough, shortness of breath, headache, fatigue, myalgia, loss of smell or taste, and sore throat). https://www.cdc.gov/coronavirus/2019-ncov/symptoms-testing/symptoms.html.

¶ https://www.unmc.edu/healthsecurity/education/programs/docs/Playbook.pdf.
